# Salinity of irrigation water selects distinct bacterial communities associated with date palm (*Phoenix dactylifera* L.) root

**DOI:** 10.1038/s41598-022-16869-x

**Published:** 2022-07-26

**Authors:** Azra Shamim, Dinesh Sanka Loganathachetti, Subha Chandran, Khaled Masmoudi, Sunil Mundra

**Affiliations:** 1 Department of Integrative Agriculture, College of Agriculture and Veterinary Medicine, United Arab Emirates University, Al-Ain, Abu-Dhabi UAE; 2 Department of Biology, College of Science, United Arab Emirates University, Al-Ain, Abu-Dhabi UAE; 3 Khalifa Center for Genetic Engineering and Biotechnology, United Arab Emirates University, Al-Ain, United Arab Emirates

**Keywords:** Ecosystem ecology, Microbial ecology, Molecular ecology

## Abstract

Saline water irrigation has been used in date palm (*Phoenix dactylifera* L.) agriculture as an alternative to non-saline water due to water scarcity in hyper-arid environments. However, the knowledge pertaining to saline water irrigation impact on the root-associated bacterial communities of arid agroecosystems is scarce. In this study, we investigated the effect of irrigation sources (non-saline freshwater vs saline groundwater) on date palm root-associated bacterial communities using 16S rDNA metabarcoding. The bacterial richness, Shannon diversity and evenness didn’t differ significantly between the irrigation sources. Soil electrical conductivity (EC) and irrigation water pH were negatively related to Shannon diversity and evenness respectively, while soil organic matter displayed a positive correlation with Shannon diversity. 40.5% of total Operational Taxonomic Units were unique to non-saline freshwater irrigation, while 26% were unique to saline groundwater irrigation. The multivariate analyses displayed strong structuring of bacterial communities according to irrigation sources, and both soil EC and irrigation water pH were the major factors affecting bacterial communities. The genera *Bacillus*, *Micromonospora* and *Mycobacterium* were dominated while saline water irrigation whereas contrasting pattern was observed for *Rhizobium*, *Streptomyces* and *Acidibacter*. Taken together, we suggest that date-palm roots select specific bacterial taxa under saline groundwater irrigation, which possibly help in alleviating salinity stress and promote growth of the host plant.

## Introduction

Date palm (*Phoenix dactylifera* L.) is an economically important tree species which is well adapted to high temperature and salinity^1,2^. It is widely cultivated in the arid environments of the Middle East and North Africa (MENA)^3^. Saline groundwater irrigation is used as a regular practice for date palm cultivation in the MENA regions^4,5^ due to water scarcity. The use of saline water for irrigation causes improper leaching in upper layers of soil, leading to salt accumulation, thereby increasing soil electrical conductivity (EC). In addition to soil EC, saline water irrigation also affects the soil organic matter (OM) and pH^6^. Irrigation with saline groundwater also impact the soil property^7^, particularly soil flocculation (Ca^2+^) or dispersion (Na^+^) depending on the ionic composition of the water^8^. Besides, higher sodium concentrations of saline water can adversely impact ionic balance of soil due to dispersal of essential cations (Ca^2+^ and Mg^2+^)^8^. Such changes in soil chemistry can potentially impact the date palm growth and their root biota.

Bacteria form complex co-associations with other microbes as well as host plant, which play a pivotal role in alleviating salinity stress as well as promoting the growth of host plant^9,10^. Root-associated bacteria (i.e. *Pseudomonas*, *Bacillus* and *Rhizobium*) are also known to accumulate osmoprotectants (i.e. choline and glycine betaine)^11–13^ and also elicit antioxidant barricading mechanisms by scavenging reactive oxygen species (ROS) under salinity stress^14^, therefore provide survival advantage to bacteria as well as host. Apart from physiological benefits, the root-associated bacteria (i.e. *Pseudomonas*, *Microbacterium* and *Arthrobacter*) are also known to promote plant growth^15^ through iron mobilization (i.e. siderophores), extracellular polysaccharides (EPS) production, phosphate solubilization and hormone production (i.e. indole acetic acid).

In a recent study, it has been shown that irrigation water salinity as a major determinant of bulk soil bacterial communities from arid agroecosystem^16^. Apart from salinity, additional factors such as soil pH and total nitrogen are also known to structure cotton field soil bacterial communities between irrigation sources (non-saline vs saline water)^6^. We also expect similar structuring of bacterial communities in date palm roots under non-saline freshwater and saline groundwater irrigation. At compositional level, saline groundwater enrich Gemmatimonadetes and Actinobacteria phyla in cotton field soil^6,17^, while barley field soil enrich specific genera namely *Rhodanobacter*, Candidatus *Koribacter* and *Burkholderia*^18^. Previous studies in date palm show compartment-specific selection of taxa^15,19^, wherein Gammaproteobacteria was dominant^19^, while another study show enrichment of *Pseudomonas* and *Rahnella* genera^20^ in the roots. Apart from the compartment effect, geographical factor (i.e. location) also affect the root-associated bacterial communities of date palm through consistant selection of Gammaproteobacteria and Alphaproteobacteria^19^ in high abundance. Furthermore, climatic factors (i.e. temperature and precipitation) are also important for structuring date palm root-associated bacterial communities^15,20^. Considering the enrichment and selection of several bacterial taxa specifically under high saline conditions of below-ground compartments of agroecosystems, we predict to have a similar response from date palm root-associated bacterial communities as well under saline groundwater irrigation.

In this study, we investigated the impact of irrigation sources (non-saline freshwater and saline groundwater) on date palm root-associated bacterial diversity and community structure. We also explored how soil and water chemistry is related with bacterial community patterns. We hypothesize that bacterial community composition while saline groundwater irrigation would be different from non-saline freshwater irrigation due to salinity stress and concomitant change in the soil chemistry. To test our hypothesis, we collected date palm root samples from different sites that receive non-saline freshwater or saline groundwater as an irrigation source and assessed the bacterial communities using 16S rDNA metabarcoding. Overall, the understanding of the date palm root-associated bacterial communities under saline conditions will help in assessing the ability to use these organisms as beneficial bio-inoculants in date palm agriculture.

## Materials and methods

### Site description, experimental setup and sampling

The study was conducted in the Al-Ain region located approximately 160 km east of Abu Dhabi (UAE; Supplementary Table S1). The region is classified under the hyper-arid category and has a mean annual rainfall of 9.03 mm and average temperature ranges between 23 °C and 35 °C in the sampling year 2020 (https://www.worldweatheronline.com/al-ain-weather-averages/abu-dhabi/ae). In our study, we selected the most commonly grown variety of date palm i.e. Khalas and sampling was performed during March 2020. The date palm trees across sites were irrigated with 50–60 L of non-saline freshwater or saline groundwater. In total, we collected 42 root samples (7 sites × 2 treatments × 3 replicates). To perform soil OM analysis, we have also collected bulk soil samples from each hole left after root excavation (approx. 10–15 cm deep). In addition, we have also collected water samples (non-saline freshwater and saline groundwater) from across sites for chemical analyses. All the samples were transported from field to lab in cooled condition, stored in − 20 °C and root samples were processed for DNA extraction within 48 h. We confirm that the use of plants in the present study complies with international, national and institutional guidelines.

### Soil and water chemistry analyses

Bulk soil samples were homogenized and passed through a 2 mm sieve. One gram of soil sample was dissolved in 9 mL of Milli-Q water and mixed for 1 h at 200 rpm. The soil–water mixture was then filtered through Whatman grade 42 filter paper and the filtrate was used for measuring soil EC and pH^21^ using Hanna pH and EC bench top meter, USA. Similarly, the irrigation water was also analyzed for EC and pH. The soil OM was analyzed using mass loss on ignition (LOI) method^22^. Briefly, 5 g of air-dried desiccated soil was incubated at 360 °C for 4 h. The difference between the mass of the soil before and after heating was used for determining the soil OM.

### DNA extraction and Illumina sequencing

The fine roots were ground with liquid nitrogen using a sterile mortar and pestle. DNA extraction was performed using one gram of the ground root tissue by DNeasy Plant Mini Kit (Qiagen, Germany) following manufacturer’s protocol. We amplified V3–V4 region of the 16S rRNA gene using 10 base pair (bp) barcoded primer combination of 341F (CCTACGGGNGGCWGCAG) and 805R (GACTACHVGGGTATCTAATCC)^23^. We set-up a 50 μL PCR reaction, consisting of forward and reverse primers (1 μM each), 250 µM dTNPs (0.5 µM of each), 0.02 U Phusion High-Fidelity DNA Polymerase (Finnzymes OY, Espoo, Finland), 0.3 mg/mL BSA (Bovine Serum Albumin) and 5× Phusion HF buffer containing 1.5 mM MgCl_2_. The applied PCR conditions were as follows: initial denaturation at 95 °C for 5 min, 25 cycles of denaturation (95 °C for 40 s), annealing (55 °C for 30 s) and extension (72 °C for 1 min), a final extension step (72 °C for 7 min). The purification and normalization of the PCR products were done using DNA Normalization Kit (Charm Biotech). The amplicon libraries were sequenced using Illumina MiSeq system (2 × 300 bp) at IMR lab, Halifax, Canada (https://www.imr.bio.com) following standard Illumina protocol. The demultiplexed raw sequence files used in this study are deposited in the Zenodo repository (10.5281/zenodo.6078292).

### Bioinformatic analyses

A total of 5,50,566 raw sequence reads were analyzed using Divisive Amplicon Denoising Algorithm 2 (DADA2_v1.12) R package (Benjamin Callahan 2016). We removed primer sequences using *rbind* function, performed quality filtering and sequence trimming (> 275 bp for forward, > 225 bp for reverse reads) (maxN = 0, truncQ = 2, maxEE = 2) using function *filterAndTrim*. The trimmed sequences were denoised using error models (*learnErrors*), and amplicon sequence variants (ASVs) were inferred for both forward and reverse reads, then contigs were generated by merging (*mergePairs*). We further removed chimera using *removeBimeraDenovo* function^24^. The ASVs were clustered into operational taxonomic units (OTUs) at 97% similarity threshold using vsearch v2.15.1^25^ as recommended in the previous study^26^. Of total 3135 OTUs (358,190 reads), 3047 non-chimeric OTUs (357,313 reads) were remained in the dataset after chimera removal using vsearch v2.15.1. These OTU sequences were processed using the same program for singleton removal, which resulted in 3040 OTUs (355,724 reads). Taxonomic assignment of the non-chimeric and non-singleton OTUs was performed using Silva v 138.1 reference database using *assignTaxonomy* function of DADA2^27^. We removed the OTUs, which were unidentified at kingdom level (20 OTUs; 229 reads), identified as Archaea (7 OTUs; 34 reads), chloroplast (10 OTUs; 169,512 reads) and mitochondria (3 OTUs; 32,008 reads), leaving 3000 OTUs (153,971 reads). We further removed nine samples with low sequence counts. Hence, 148 OTUs (5607 reads) strictly present in those samples were also filtered. The final OTU table after performing afore-mentioned steps consisted of 2852 OTUs (148,904 reads). Prior to diversity analyses using R package *vegan*, OTU table was normalized based on sample with lowest number of sequences (625 reads) using R function *rrarefy*. The OTUs were classified as abundant (> 1%), moderate (0.1–1%) and rare taxa (< 0.1%) based on % occurrences according to a previous study^28^.

### Statistical analyses

All the statistical analyses were performed in R v4.0.3 (R Core Development Team, 2020) using respective packages unless stated otherwise. To perform community structural analyses, OTU abundance data was arcsine-transformed to increase the homogeneity of variances. Water and soil chemistry variables including pH, EC and soil OM were standardized using Z transformation and expressed on a 0–1 scale, which helps in the comparison of values from disparate distributions. To examine the effects of irrigation source (non-saline freshwater vs saline groundwater) on soil (pH, EC and OM) and water chemistry (pH and EC) variables, we used analyses of variance (ANOVA) test, followed by Tukey’s HSD post-hoc test using R package *agricolae*. The between site variation of environmental metadata was performed using analyses of variance (ANOVA) test, followed by Tukey’s HSD pair-wise test using R package *agricolae.* The impact of irrigation sources (non-saline freshwater vs saline groundwater) on bacterial richness, Shannon diversity index and Pielou’s evenness index was also analyzed using similar statistical test. Linear regression analyses were performed using R package *vegan*, to investigate the relationship of soil and water chemistry variables with bacterial richness, Shannon diversity index and Pielou’s evenness index (*P* < 0.05)^29^.

To perform multivariate analyses, we calculated dissimilarities in OTUs matrices using Bray–Curtis distances. The relationship of irrigation sources (non-saline freshwater vs saline groundwater), soil (pH, EC and OM) and water (pH and EC) chemistry variables in explaining bacterial community structuring was assessed by permutational analysis of variance (PERMANOVA) using *Adonis* function *vegan*. We calculated pseudo-F statistics following 9999 times permutation of the OTUs matrices. A forward selection procedure was exercised to optimize the final model for PERMANOVA analyses^30^. At first, we tested single factor models, and thereafter significant factors were added in the order of their R^2^ values to obtained final significant model. To investigate bacterial community structural patterns, we also performed Nonmetric Multi-Dimensional Scaling (NMDS) ordination analysis using *metaMDS* function of *vegan*. NMDS was performed with following settings, dimensions (k) = 2, maximum iterations = 1000, initial configurations = 100, minimum stress improvement in each iteration cycle = 10^–5^ and *P* value and R^2^ values were calculated. Bacterial community structural relationship with irrigation water sources, soil and water chemistry variables were inferred using *envfit* function of *vegan* and vector variables were fitted on NMDS ordination space.

## Results

### Sequence data characteristics

The mean reads per sample was 4512 (range 625–16,252) and the mean OTU richness per sample was 52 (range 2–5283) (Supplementary Fig. S1). The 20 most abundant OTUs accounted for 30% of the total reads. Overall, Proteobacteria (37.4%) and Actinobacteria (28.2%) were the most common phyla detected in date palm roots followed by Firmicutes (10.1%), Chloroflexi (5.4%), Acidobacteriota (4.8%) and Bacteroidota (4.0%) (Supplementary Table S2).

### Saline groundwater irrigation effect on soil chemistry and bacterial diversity

We found that soil OM and irrigation water pH as well as EC differed significantly between non-saline freshwater and saline groundwater irrigation (Fig. S2). Irrigation water pH and soil OM were significantly higher in the samples where non-saline freshwater was the irrigation source, and pattern contrasted for irrigation water EC.

**Figure 1:**
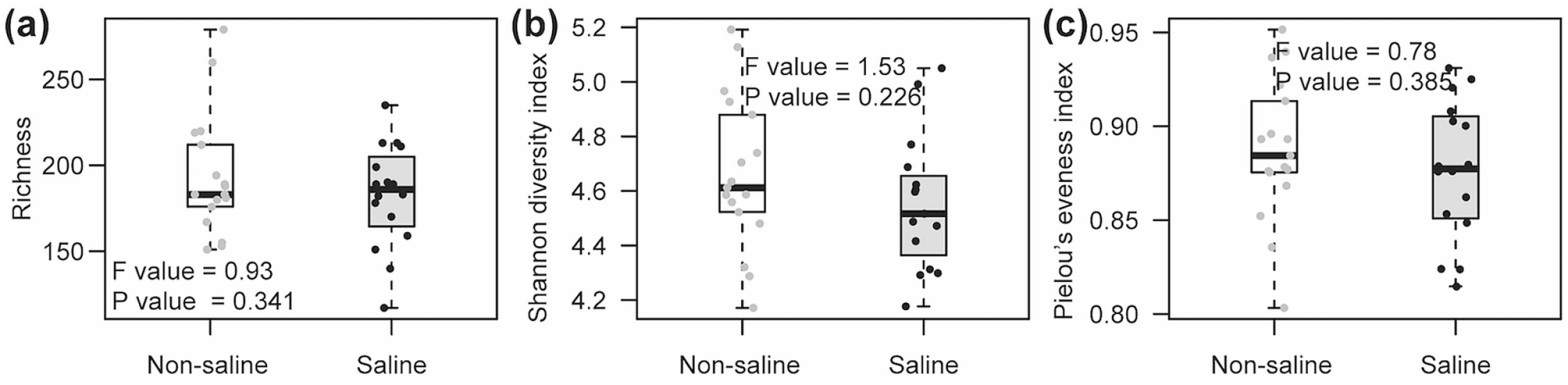
Effect of irrigation sources (non-saline freshwater vs saline groundwater) on date palm root-associated bacterial alpha diversity. Irrigation source effect on (**a**) bacterial richness; (**b**) Shannon diversity index; (c) Pielou’s evenness index. Statistical inference is highlighted within each panel of the plot and assessed using ANOVA analyses followed by Tukey’s HSD post hoc test (*P* < 0.05). The box spans the interquartile range (IQR; first quartile to the third) with the median indicated by a dark horizontal line, the whiskers show the 1.5× IQR. Data for each sample is also displayed with strip chart.

**Figure 2:**
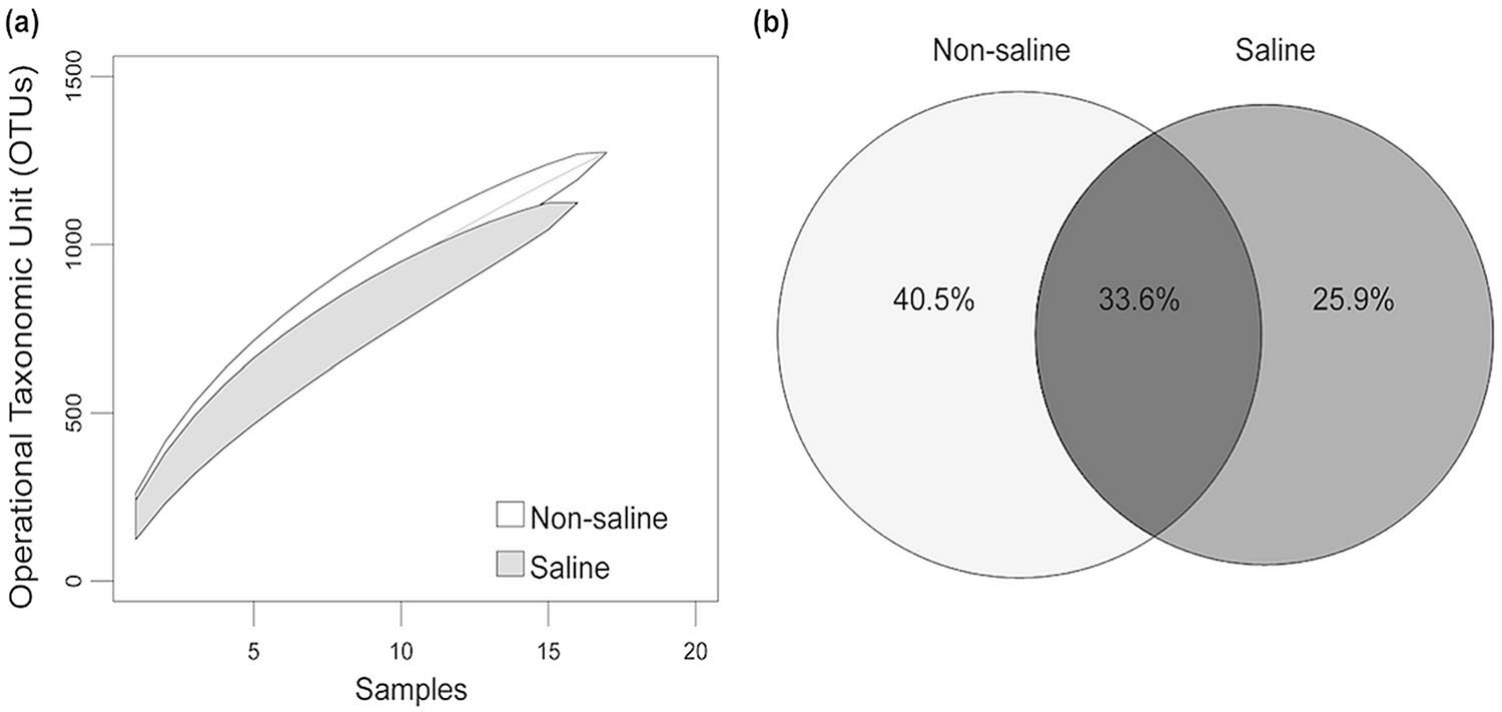
The species accumulation curves, unique and shared bacterial OTU analysis. (**a**) Operational taxonomic unit (OTU) accumulation curves at 97% similarity and (**b**) shared and unique OTUs of date palm root between irrigation sources (non-saline freshwater vs saline groundwater). The unique and shared OTUs are expressed as percentages of total OTUs (2852).

**Figure 3:**
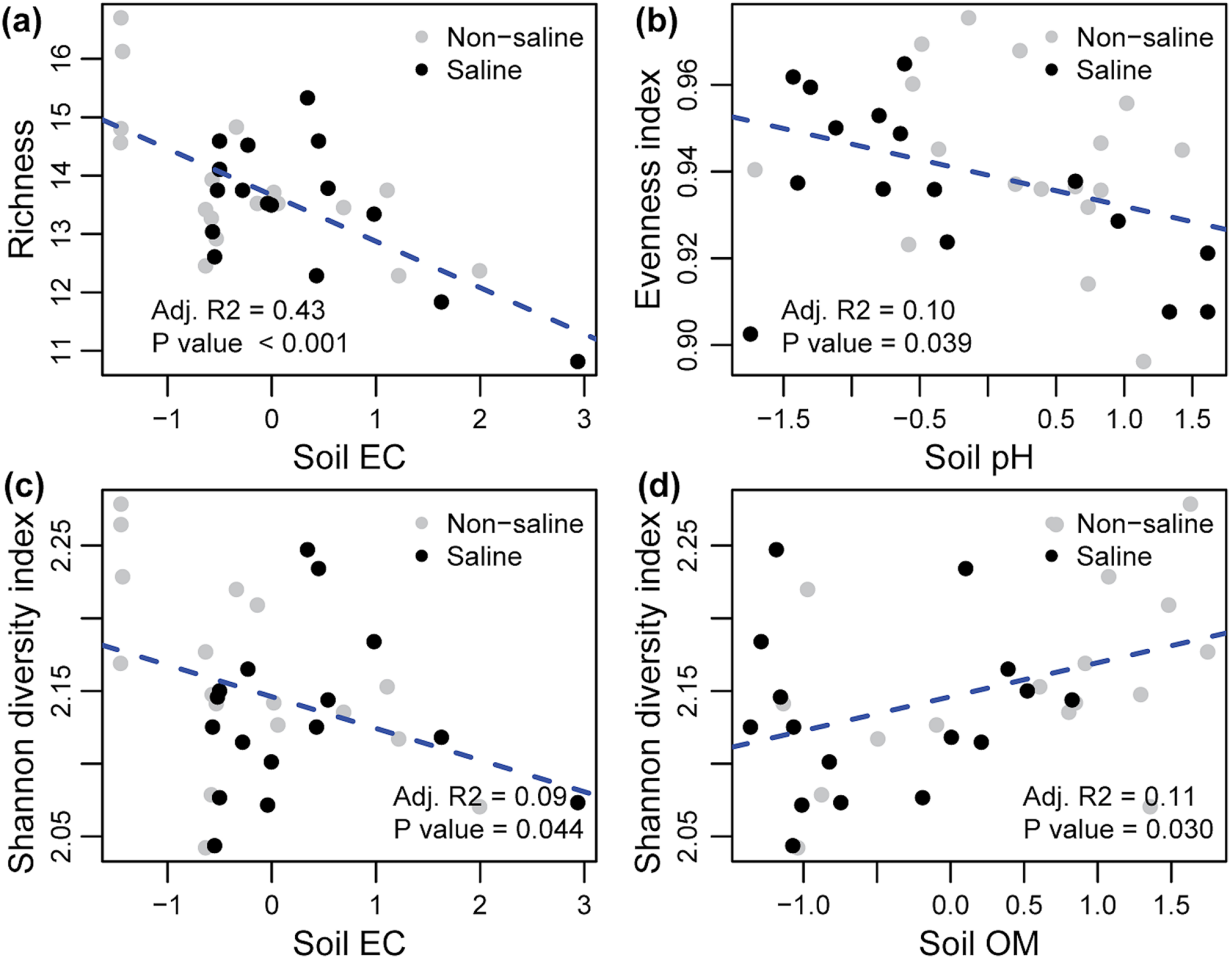
Linear regression analyses between bacterial diversity measures and soil chemistry. Relationship between (**a**) bacterial richness; (**b**) evenness index; and (**c,d**) Shannon diversity index with environmental metadata. The statistical inference (R^2^ and *P* values) and regression line (blue colour) are highlighted within each panel. *EC* electrical conductivity (dS/m), *OM* organic matter (%).

The saline groundwater irrigation did not affect bacterial diversity including richness, Shannon diversity index and Pielou’s evenness index (Fig. 1a–c). However, the OTU accumulation curve displayed presence of relatively higher number of total OTUs in the samples where non-saline freshwater was used as a source of irrigation (Fig. 2a). 40.5% of total OTUs were exclusively detected in roots of date palm under non-saline freshwater irrigation and 26% were unique to saline groundwater irrigation (Fig. 2b). Only 33.6% were shared between both irrigation sources. We also found that all the unique bacterial OTUs detected under saline groundwater irrigation belonged to rare taxa (< 0.1% occurrences) (Supplementary Table S3). The linear regression analyses showed that the bacterial richness and the Shannon diversity index were negatively correlated soil EC (Fig. 3a,b). Similarly, the Pielou’s evenness index also showed negative correlation to soil pH (Fig. 3c). Shannon diversity index also showed a significant positive correlation with the soil OM concentrations (Fig. 3d). The between site variations of water and soil chemistry is given in Supplementary Tables S3 and S4. Non-saline water irrigation showed highest soil pH in site 5, meanwhile highest soil EC was observed in site 2. Saline water irrigation showed highest soil pH and soil OM in site 4, while site 5 recorded highest soil EC.

### Saline groundwater irrigation affects bacterial community structural patterns

**Figure 4:**
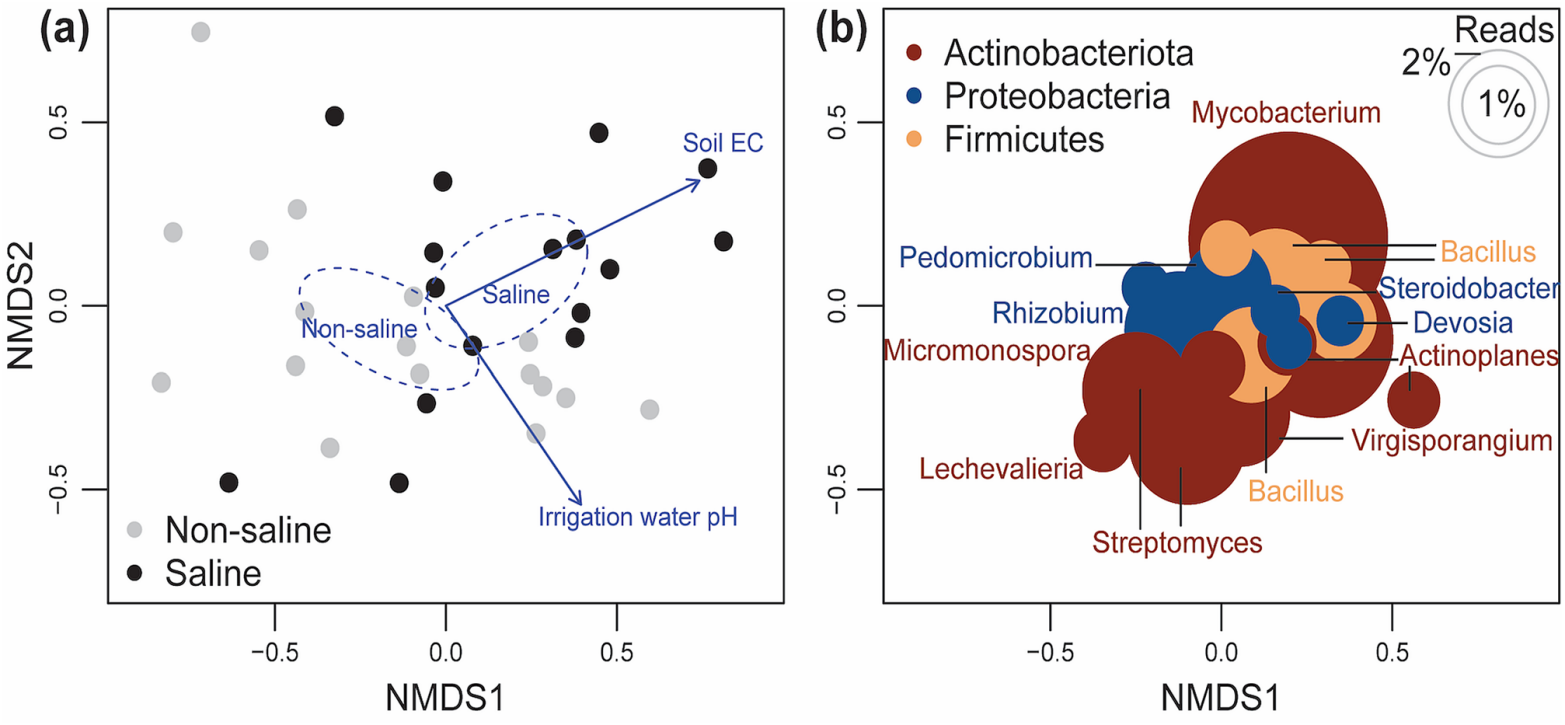
Non-metric multidimensional scaling (NMDS) ordination analysis for date palm roots-associated bacterial communities between irrigation sources (non-saline freshwater vs saline groundwater). (**a**) The sample-based ordination plot was generated from total Operational Taxonomic Units (OTUs) compositions. The different colours in a (**a**) are coded according to irrigation source, and in a panel, (**b**) coded according to bacterial phyla. The ellipse represents 95% confidence interval for the tested factor variable (i.e., irrigation source), and arrows point in the direction of maximum increase of individual vector variables and had significant effects (*P* < 0.05) on the ordination configuration. (**b**) Species plots of overall bacteria was also based on total OTUs composition, but the most common 20 OTUs are visualized. The size of the circles in panel b shows the relative abundance of the OTUs.

The multivariate methods, including PERMANOVA and NMDS ordination analyses, indicated the presence of distinct root-associated bacterial communities between different irrigation sources i.e. non-saline freshwater and saline groundwater (R^2^ = 0.06, *P* = 0.002) (Fig. 4, Table 1).Two distinct clusters belonging to non-saline freshwater and saline groundwater irrigation was observed in the NMDS ordination space indicating presence of distinct bacterial communities when irrigation source differ. Further, the PERMANOVA analyses indicated that soil EC (R^2^ = 0.07, *P* = 0.001) and water chemistry [pH (R^2^ = 0.05, *P* = 0.006) and EC (R^2^ = 0.05, *P* = 0.015)] were the significant factors affecting bacterial community structural patterns.

**Table 1:** PERMANOVA analysis showing effects of different irrigation sources (non-saline vs saline water) on date palm root-associated bacterial community compositional structure.

Source of variation	Df	SumsOfSqs	MeanSqs	F.Model	R^2^	Pr(> F)
EC (soil)	1	0.53	0.53	2.53	0.07	** < 0.001**
pH (irrigation water)	1	0.40	0.40	1.89	0.05	**0.006**
Irrigations	1	0.45	0.45	2.13	0.06	**0.002**
EC (irrigation water)	1	0.36	0.36	1.72	0.05	**0.015**
Residuals	28	5.88	0.21	0.77		
Total	32	7.62	1			

Effects of irrigation source, irrigation water pH, irrigation water EC (dS/m); soil (pH, EC, and organic matter (OM) chemistry were tested on overall bacterial communities.

P‐values were obtained using 9999 permutations, and boldface indicates statistical significance (*P* < 0.05).

### Saline groundwater irrigation effect on bacterial community composition

**Figure 5:**
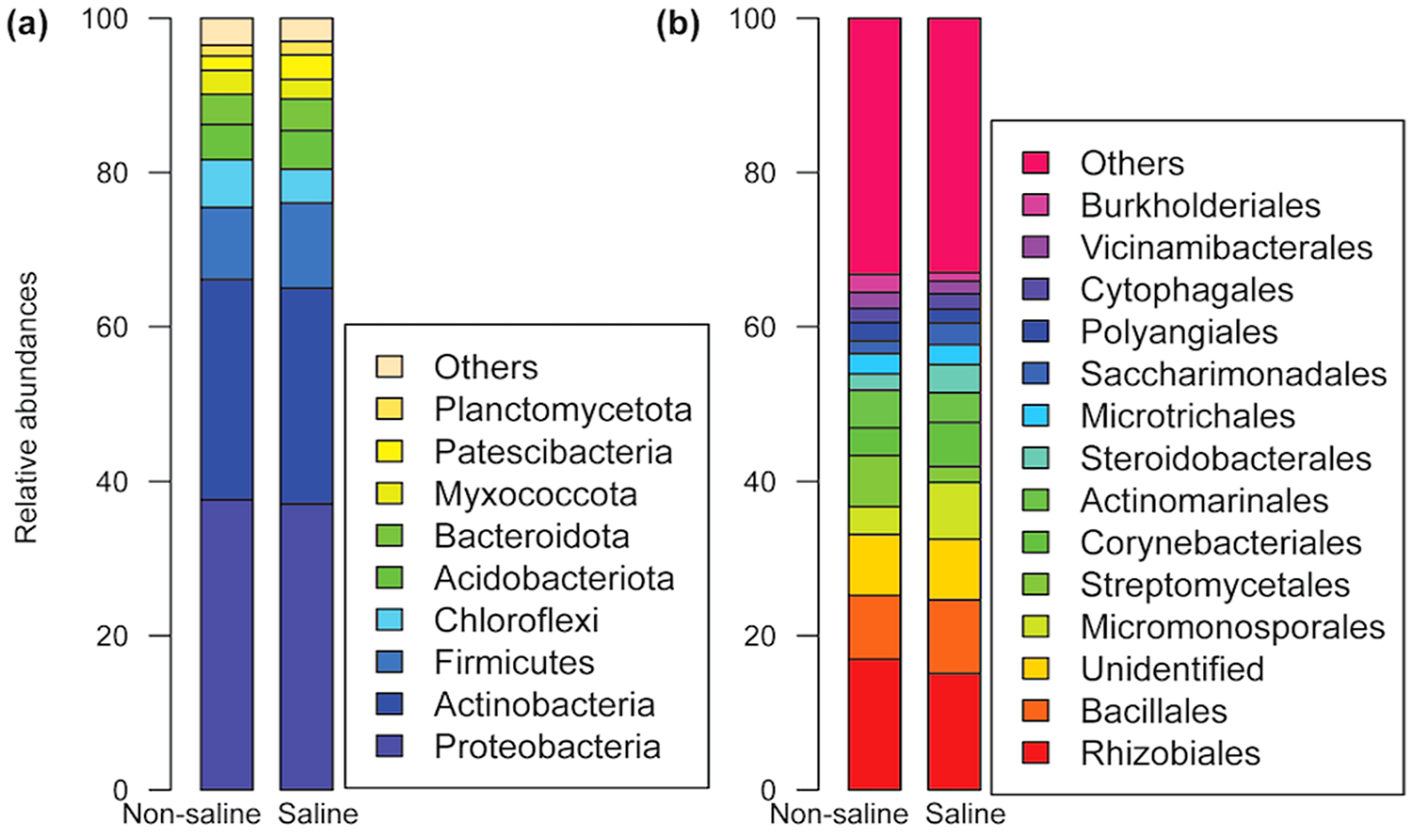
The relative abundance distribution of top taxa. The relative abundance at (**a**) phylum and (**b**) order levels of date palm root treated with different irrigation source (non-saline freshwater vs saline groundwater).

**Figure 6:**
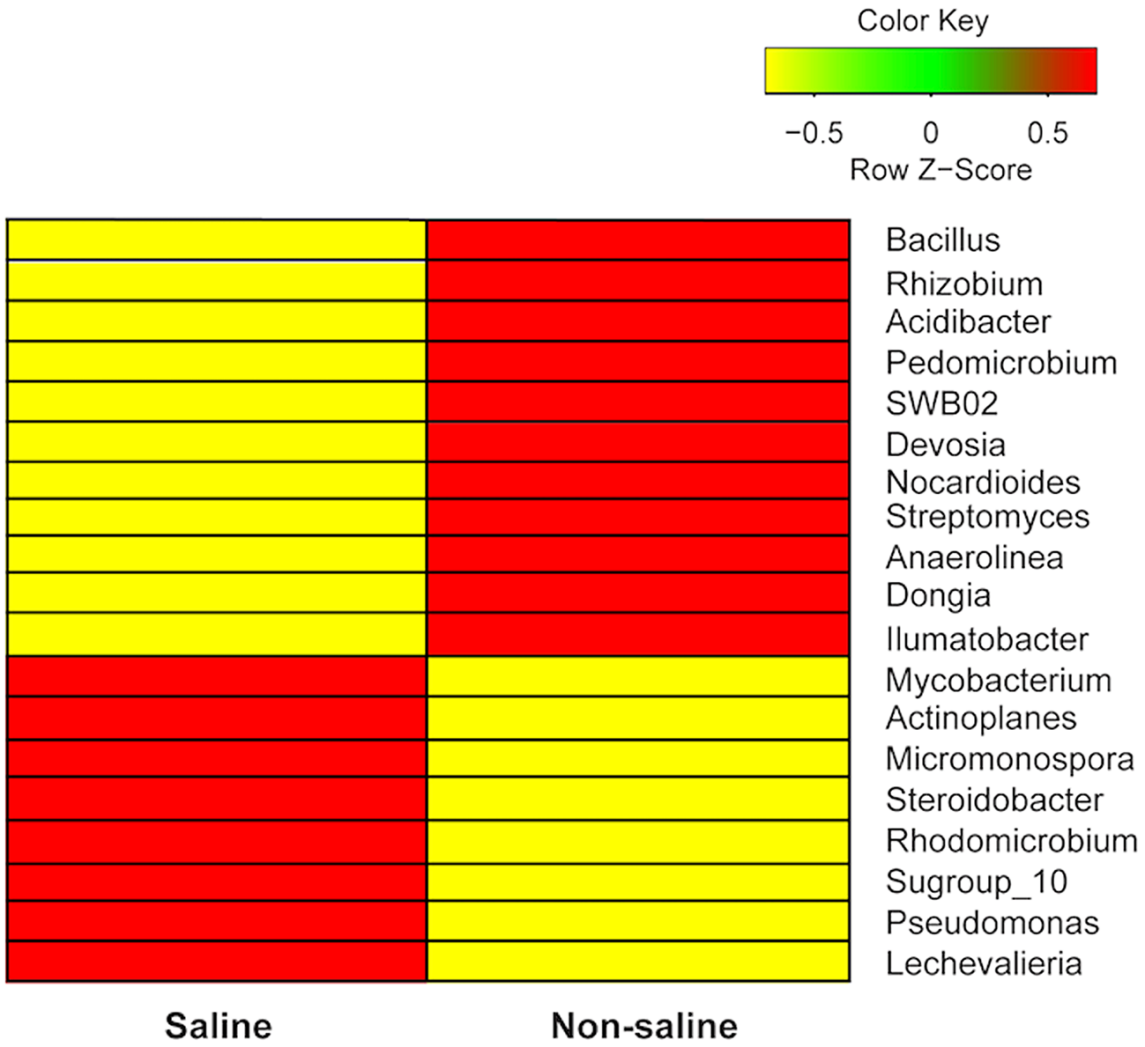
Heat-plots for proportional abundances of date palm root-associated bacterial genera under non-saline freshwater and saline groundwater irrigation. The figure shows hierarchical clustering of significant OTUs (*P* < 0.05) of date palm roots between irrigation sources (saline groundwater vs non-saline freshwater). The legend with colour key indicates median-centered Z-scores, which were calculated after normalizing relative abundance values of selected genera.

Phylum Chloroflexi was relatively more abundant in root samples where non-saline freshwater was the irrigation source and abundance patterns were contrasted for the phylum Firmicutes, being more common while saline groundwater irrigation (Fig. 5a, Supplementary Table S2). At order level, we found that relative abundance of Rhizobiales, Streptomycetales, Actinomarinales, and Burkholderiales was higher while non-saline freshwater irrigation compared to saline groundwater and opposite patters were observed for orders Bacillales, Micromonosporales, Corynebacteriales, and Steroidobacterales (Fig. 5b). The hierarchical clustering analysis using proportional abundances of bacterial genera revealed that *Bacillus*, *Rhizobium*, *Acidibacter* and *Streptomyces* were more abundant in the sample having non-saline freshwater irrigation (*P* < 0.05) (Fig. 6, Table 2). Similarly, bacterial genera such as *Mycobacterium*, *Micromonospora*, *Steroidobacter* and *Pseudomonas* were abundant while saline groundwater irrigation (*P* < 0.05) (Fig. 6, Table 2). The most common OTUs with taxonomic affinity to *Actinocorallia, Bacteroides* and SWB02 were more abundant in the samples when non-saline freshwater was the irrigation source whereas Thermoanaerobaculaceae subgroup 10, *Chryseobacterium*, *Rheinheimera and Blastocatella* were richer while saline groundwater irrigation (Supplementary Table S4).

**Table 2:** Taxonomic affinity, read abundance and occurrences of the 20 most abundant operational taxonomic units (OTUs) detected from date palm roots under different irrigation source (saline vs non-saline water).

OTU ID	Genus (Phylum)**	Overall		Non-saline		Saline	
		Reads (%)	Occurances (%)*	Reads (%)	Occurances (%)^#^	Reads (%)	Occurances (%)^$^
OTU_3	Mycobacterium (A)	3.5	100.0	2.9	94.1	6.1	100.0
OTU_6	Unidentified (A)	2.6	96.9	3.5	88.2	2.7	100.0
OTU_10	Streptomyces (A)	2.1	93.8	3.2	100.0	1.7	81.3
OTU_13	Rhizobium (P)	2.0	100.0	2.4	100.0	2.3	93.8
OTU_28	Micromonospora (A)	1.9	84.4	1.6	94.1	3.2	68.8
OTU_143	Streptomyces (A)	1.8	78.1	3.4	82.4	0.4	68.8
OTU_4	Unidentified (F)	1.7	90.6	1.7	94.1	2.5	81.3
OTU_21	Pedomicrobium (P)	1.6	103.1	2.0	100.0	1.9	100.0
OTU_9	Bacillus (F)	1.6	78.1	2.7	76.5	0.8	75.0
OTU_15	Bacillus (F)	1.3	78.1	1.1	70.6	2.3	81.3
OTU_7	Actinoplanes (A)	1.2	65.6	0.5	70.6	2.6	56.3
OTU_16	Virgisporangium (A)	1.0	93.8	0.6	94.1	2.1	87.5
OTU_12	Lechevalieria (A)	1.0	59.4	0.6	64.7	2.1	50.0
OTU_8	Actinoplanes (A)	0.9	56.3	1.4	47.1	0.7	62.5
OTU_23	Bacillus (F)	1.0	84.4	1.1	82.4	1.2	81.3
OTU_84	Unidentified (F)	0.9	93.8	0.9	94.1	1.4	87.5
OTU_36	Unidentified (P)	0.9	100.0	1.1	94.1	1.0	100.0
OTU_24	Unidentified (P)	0.9	100.0	1.3	100.0	0.7	93.8
OTU_38	Steroidobacter (P)	0.8	84.4	0.8	70.6	1.4	93.8
OTU_25	Devosia (P)	0.8	84.4	1.2	82.4	0.6	81.3

Total reads (%) and occurrences among samples were calculated for overall database, non-saline sample and saline samples subset.

**Abbreviation (A), (P) and (F) represent bacterial phyla Actinobacteria, Proteobacteria and Firmicutes, respectively.

*Occurrence (%) calculated from total 33 samples.

^#^Occurrence (%) calculated from total 17 non-saline samples.

^$^Occurrence (%) calculated from total 16 saline samples.

## Discussion

We find that date palm root-associated bacterial diversity does not change but number of unique bacterial OTUs associated with date palm roots vary under different irrigation sources. Saline groundwater irrigation strongly alters the bacterial community structure and soil EC as well as irrigation water pH are the major factors affecting their pattern. Abundance of genera *Rhizobium*, *Streptomyces* and *Acidibacter* is higher under non-saline freshwater irrigation, whereas *Bacillus*, *Micromonospora, Pseudomonas* and *Mycobacterium* are more common in the samples with saline groundwater irrigation. We didn’t assess the role of geo-climatic factors (i.e. MAT and MAP) since focus of our study is to understand saline groundwater irrigation impact on root-associated bacterial communities, but they may also act as possible factor(s) of soil community structure across sites in this study.

### Saline groundwater irrigation altered soil OM

The irrigation water source (non-saline freshwater and saline groundwater) did not significantly affect soil EC and pH (Fig. S2a,b) across sites in our study, which may attribute to rapid percolation of water through soil layers since soil types (i.e. Entisol and Aridsol) prevalent in arid agroecosystems of UAE^31^ are known to have lower water holding capacity. On contrary, the irrigation water source altered soil OM between treatments (Fig. S2c), the decrease in soil OM concentrations under saline groundwater irrigation is possibly primed by root exudates which enhance bacterial degradation of soil OM through rhizosphere priming effect^32^. In addition, saline groundwater can also potentially affect the quantities of root-derived carbon^33^, which is evident from decreased soil OM.

### Saline groundwater irrigation changes community structure patterns but not diversity

We found that saline groundwater irrigation affected the date palm root-associated bacterial community structural patterns but not the diversity including richness, Shannon diversity index and Pielou’s evenness index. Although diversity didn’t change but we observed altered presence of unique OTUs while irrigating date-palm with different water sources indicating selection of specific bacterial species. Moreover, the entire set of unique OTUs detected under saline groundwater irrigation of this study belonged to rare taxa. Therefore, irrigation water source driven changes in unique OTUs and their proportions possibly structured the bacterial communities. Our results are in agreement with the previous studies, reporting irrigation source specific distinct bacterial communities in plants (i.e. cotton and barley) grown in arid environment^6,17,18^. The observed community structural pattern can be explained by a deterministic salinity filtering process, wherein the selection of bacterial assemblages depend on the salinity tolerance of date palm roots. The plant roots under salinity stress can act as “gatekeepers” by selecting soil bacteria from the rhizosphere and salinity trait may play a critical environmental filter in selectively enriching bacterial species in root niches. The salinity filtering is perhaps governed by high salt concentration, which could affect the survival and replication of bacteria in root through plasmolysis^34^.

Soil EC and irrigation water pH were the most important factors affecting root-associated bacterial community structural patterns. Firstly, salinity (which is an indirect measure of soil EC) is one of the key determinants for soil microbial communities in a desert ecosystem^35^ and it is known to affect desert plant root-associated bacterial communities^18,35^. The soil EC mediated alteration in bacterial taxa can potentially influence nutrient availability since salinity and nitrogen concentrations are reportedly inter-connected in the rhizosphere^36^. Increase in soil EC negatively impacted bacterial richness and Shannon diversity index, indicating bactericidal effect of salinity^34^ as reported by a previous study on desert plants irrigated with high-saline water^17^. Secondly, the irrigation water pH is known to regulate bacterial communities either directly and/or indirectly by affecting the availability of cations or anions in the plant rhizosphere^37^. The relative increase in irrigation water pH under non-saline freshwater irrigation could be contributed by lime dissolution associated increase in calcium levels^38^, which in turn possibly structured bacterial communities by selecting bacteria depending on their pH preference. The alterations in irrigation water pH also affects the soil pH due to water flow and percolation in soil layers. Moreover, pH of soil was reported as one of the major predictors of bacterial diversity^39,40^ and its increase negatively affected evenness of root-associated bacteria in this study is in line with a previous study^38^.

### Enrichment of specific bacterial taxa by date palm roots

The pattern of distribution of the major phyla Proteobacteria, Actinobacteria and Acidobacteriota was similar in roots under both types of irrigation water sources. The higher abundance of these phyla seems to be common for plants (i.e. cotton and barley) while growing in desert environments under different irrigation water salinities ^6,17^, which play a vital role in ROS homeostasis and maintain ionic balance in the roots of date palm, therefore indirectly supports host plant under drought conditions^41^. In addition, these phyla also play an important role in nitrogen fixation (Actinobacteria and Proteobacteria), decomposition of soil OM (Actinobacteria) and biological control (Actinobacteria and Acidobacteriota)^42–44^. We found higher dominance of Chloroflexi in date palm roots under non-saline freshwater irrigation, which are important in breaking down complex carbohydrates and polymeric organic compounds into low molecular weight substances within the rhizosphere^45^. On contrary, Firmicutes was relatively higher in saline groundwater irrigation, whose members are known to be involved in salinity stress tolerance^46^. We found lower dominance of the following orders, Rhizobiales, Streptomycetales, Actinomarinales and Burkholderiales and their representative genera in roots under non-saline freshwater irrigation indicate their aversion to higher salinity. Rhizobiales was represented by *Rhizobium* in roots under non-saline freshwater irrigation, a fast growing rhizobacteria^47^ observed generally in the nodules of leguminous plants^48^. Previous studies show that non-leguminous date palms also contain *Rhizobium* in the rhizosphere^15,19^, which is possibly involved in the promotion of plant growth^49^. The reason for the higher abundance of *Rhizobium* in roots of non-saline freshwater irrigation may be due to lack of salinity stress and higher soil OM, which possibly enabled *Rhizobium* to multiply faster in roots. The abundance of *Streptomyces* was also higher in roots under non-saline freshwater irrigation, which is known to carry out anti-phytopathogenic and biocontrol roles in the plant rhizosphere^50^. *Acidibacter* detected in the roots of non-saline freshwater irrigation, is an acidophilic iron-reducing bacterium^51^ possibly involved in contributing iron to roots.

In contrary, bacterial species found in this study and belonging to Bacillales, Micromonosporales, Corynebacteriales and Steroidobacterales were more common under saline groundwater irrigation, which is in line with previous reports on plants growing under saline conditions^52–55^. Bacillales consisted of *Bacillus* genus*,* which produces spores for survival under saline stress conditions^56,57^, is also capable of producing rhizosheath made up of EPS around plant roots and confer salinity resistance by excluding Na^+^ ions from plant roots^55^. Another saline groundwater irrigation specific order detected was Micromonosporales represented by *Micromonospora* genus, which is known to perform complex polysaccharide degradation^58^, mobilize nutrients for the plant through metallophores^59^ and perform plant growth promotion through aminocyclopropane-1-carboxylic acid (ACC) deaminase activity^60^. The *Micromonospora* is also reported to contain genes related to osmotic stress tolerance (*betC* and *proU*) and plant growth promotion (indole-3-glycerol phosphate synthase)^61^. The other dominant genera *Pseudomonas* and *Mycobacterium* detected in this study were also earlier reported in the plant rhizosphere^19,62^. The *Mycobacterium* genus is known to perform plant growth promoting activity under high temperature and hyper-saline conditions^62^, thus providing an advantage to the survival of date palms under salinity stress. The *Pseudomonas* genus was one of the major genera in the date palm rhizosphere^19^ in this study, is known to provide biocontrol properties and increase plant fitness by triggering salicylic acid-mediated systemic acquired resistance (SAR) in plants^63^. The other dominant OTUs *Chryseobacterium* and *Blastocatella* were detected only in date palm roots under saline groundwater irrigation, are known to perform plant growth promotion (ACC deaminase, siderophore, ammonia and hydrogen cyanide production) under saline stress conditions^64,65^. These results suggest that date palm roots select a set of bacterial species at higher abundance depending on irrigation water sources (non-saline freshwater vs saline groundwater). These selected taxa potentially help host plants to allevite salinity-induced stress and increase plant growth and performance.

## Conclusion

Our findings show that saline groundwater irrigation does not affect date palm root-associated bacterial diversity, but alter compositional patterns, which is mainly affected by soil EC and irrigation water pH. The enrichment of taxa (*Bacillus*, *Micromonospora*, *Mycobacterium* and *Pseudomonas*) under saline groundwater irrigation revealed how date palm roots adapt to salinity by selectively enriching specific bacteria. The increased abundance of these salinity resistant bacteria under saline groundwater irrigation is very important for the survival of the date palms under drought conditions due to their potential role in plant growth promotion and nutrient mobilization. Overall, this study revealed that saline groundwater induce perturbations in enrichment or reduction of certain bacterial species, which may potentially help the host plant to alleviate salinity stress.

## Supplementary Information

Supplementary Information.

## Data Availability

Raw sequence data is submitted in Zenodo repository (10.5281/zenodo.6078292).
