# Supplementary material for: Salinity of irrigation water selects distinct bacterial communities associated with date palm (Phoenix dactylifera L.) root
Source: Sci Rep. 2022 Jul 26;12:12733. doi: 10.1038/s41598-022-16869-x (PMC9325759; doi:10.1038/s41598-022-16869-x)
Supplement: Supplementary file 1 — Supplementary Information. [file 41598_2022_16869_MOESM1_ESM.docx]

***Supplementary Information for***

***Salinity of irrigation water selects distinct bacterial communities associated with Date palm (Phoenix dactylifera L.) root***

**Azra Shamim^1, #^, Dinesh Sanka Loganathachetti^2, #^, Subha Chandran^2^, Khaled Masmoudi^1,*^, Sunil Mundra^2,*^**

^1^Department of Integrative Agriculture, College of Agriculture and Veterinary Medicine, United Arab Emirates University, Al-Ain, Abu-Dhabi, UAE.

^2^Department of Biology, College of Science, United Arab Emirates University, Al-Ain, Abu-Dhabi, UAE.

***# Shared first author***

## *Corresponding author:

**Khaled Masmoudi**

Email : khaledmasmoudi@uaeu.ac.ae

**Sunil Mundra**

E-mail: [sunilmundra@uaeu.ac.ae](mailto:sunilmundra@uaeu.ac.ae)

Phone: +971-03-7136341


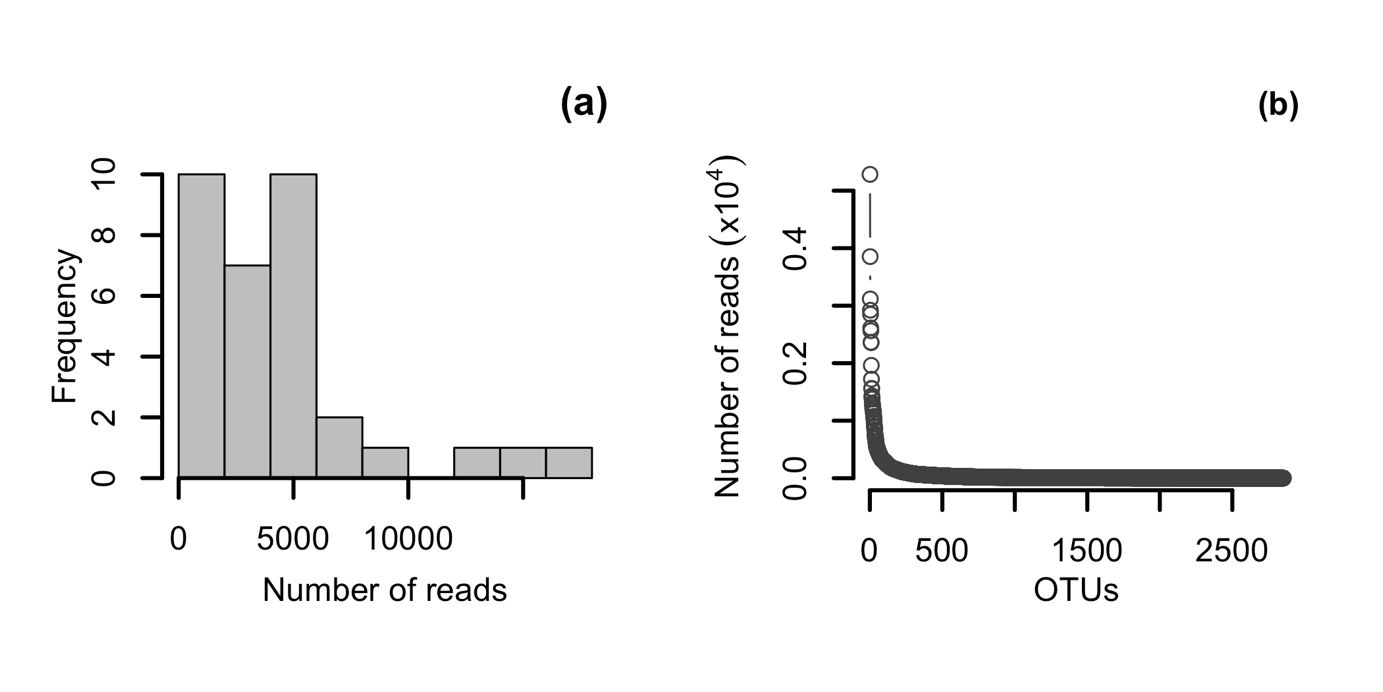


**Figure S1.** Sequence data characteristics for overall bacterial dataset. (a) Bar plots displaying distribution of reads per sample; and (b) rank-abundance plot demonstrating the number of reads per OTU.

**
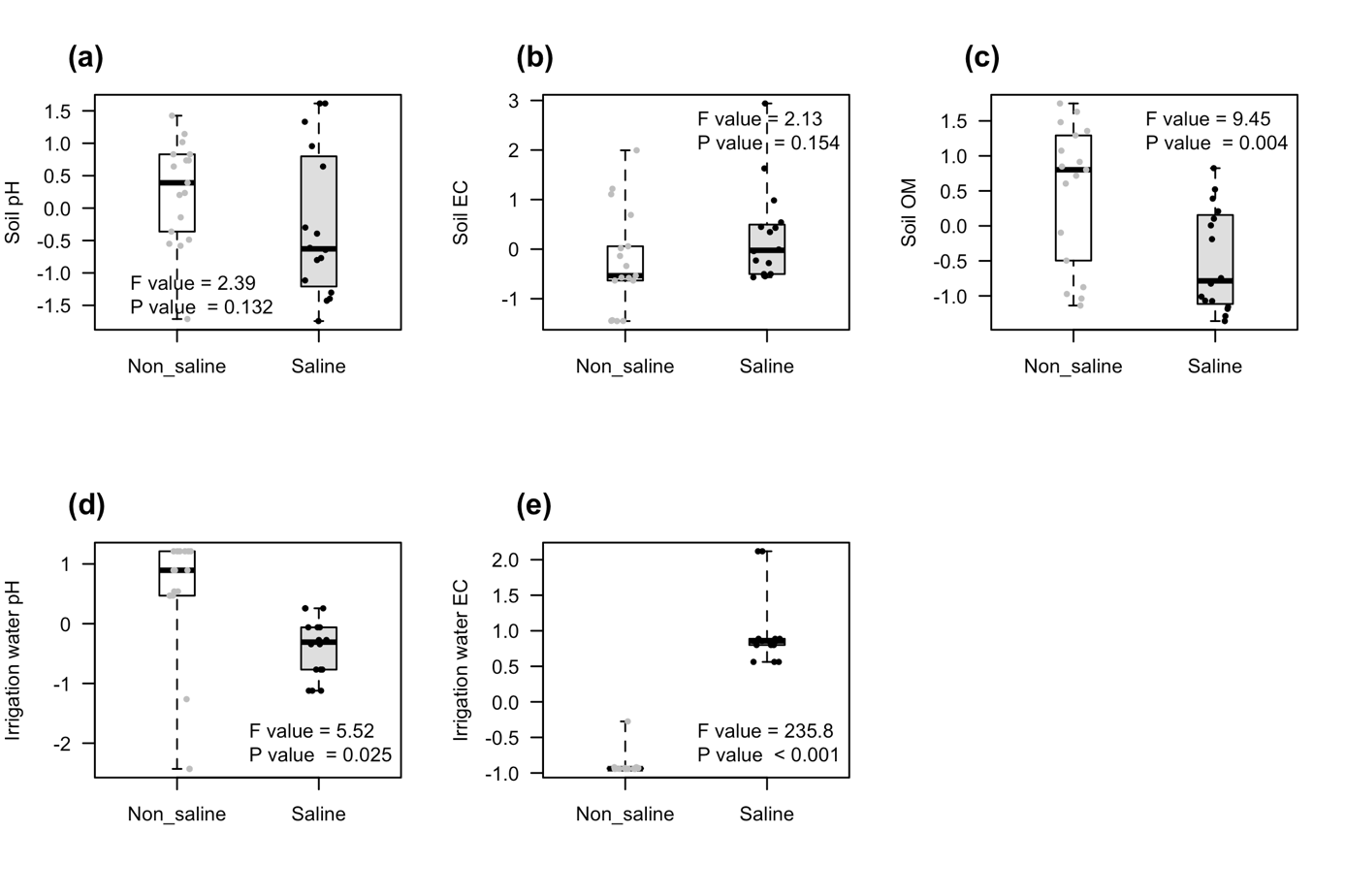
**

**Figure S2.** Box plots showing the environmental metadata (a) soil pH; (b) soil electrical conductivity (EC in dS/m); (c) soil organic matter (OM); (d) irrigation water pH; and (e) irrigation water EC (EC in dS/m), measured in this study under different irrigation water sources (non-saline freshwater vs saline groundwater) conditions. Statistical inference is highlighted within each panel of the plot and assessed using ANOVA analyses followed by Tukey’s HSD post hoc test (*P*< 0.05). The box spans the interquartile range (IQR; first quartile to the third) with the median indicated by a dark horizontal line, the whiskers show the 1.5xIQR. Data for each sample is also displayed with strip chart.

# **
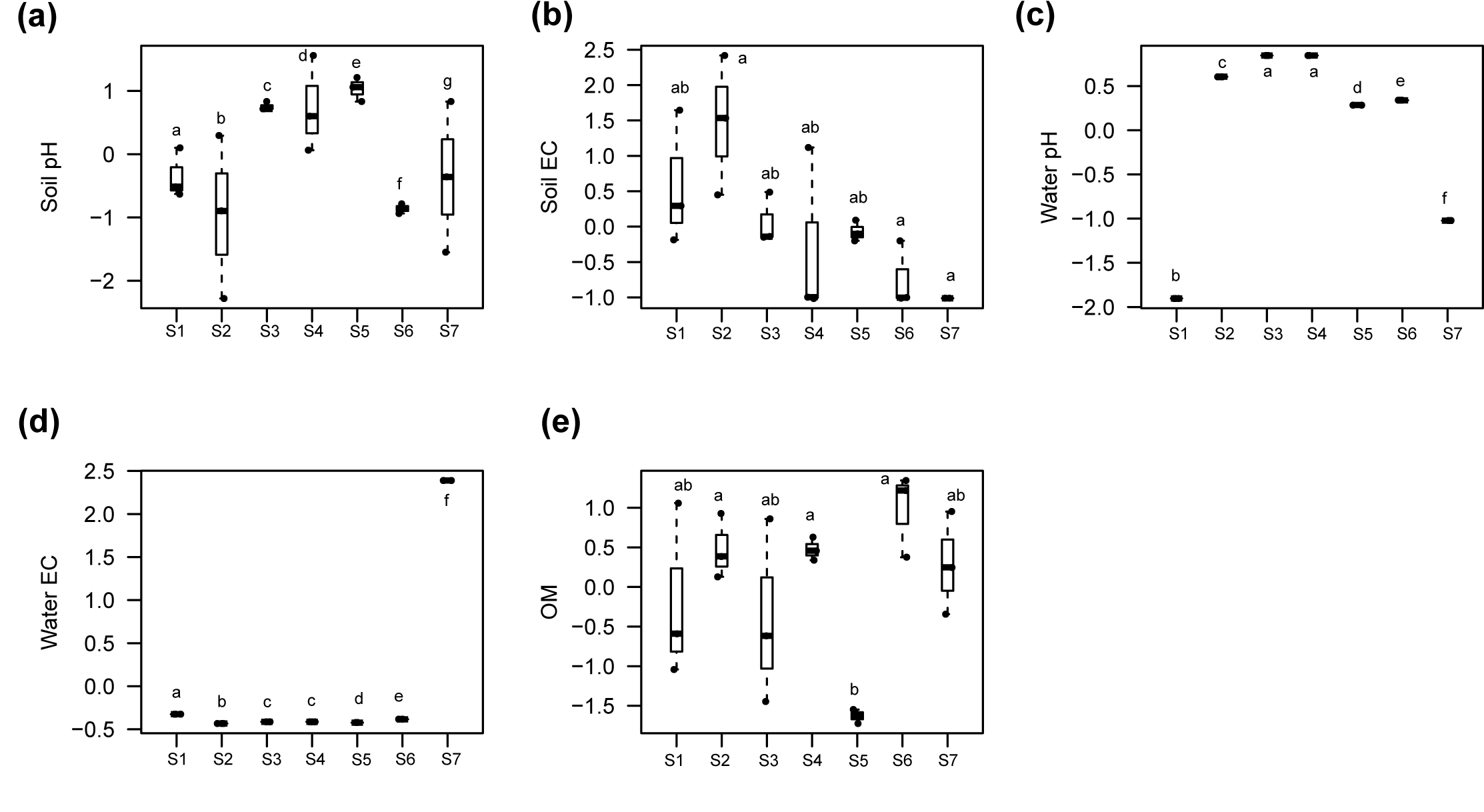
**

**Figure S3.** Box plots showing the environmental metadata under non-saline freshwater irrigation (a) soil pH; (b) soil electrical conductivity (EC in dS/m); (c) soil organic matter (OM); (d) irrigation water pH; and (e) irrigation water EC (EC in dS/m), measured in this study under different irrigation water sources (non-saline freshwater vs saline groundwater) conditions. Statistical inference is highlighted within each panel of the plot and assessed using ANOVA analyses followed by Tukey’s HSD pair-wise test. The different alphabets denote significance at 0.05. The box spans the interquartile range (IQR; first quartile to the third) with the median indicated by a dark horizontal line, the whiskers show the 1.5xIQR. Data for each sample is also displayed with strip chart.

# **
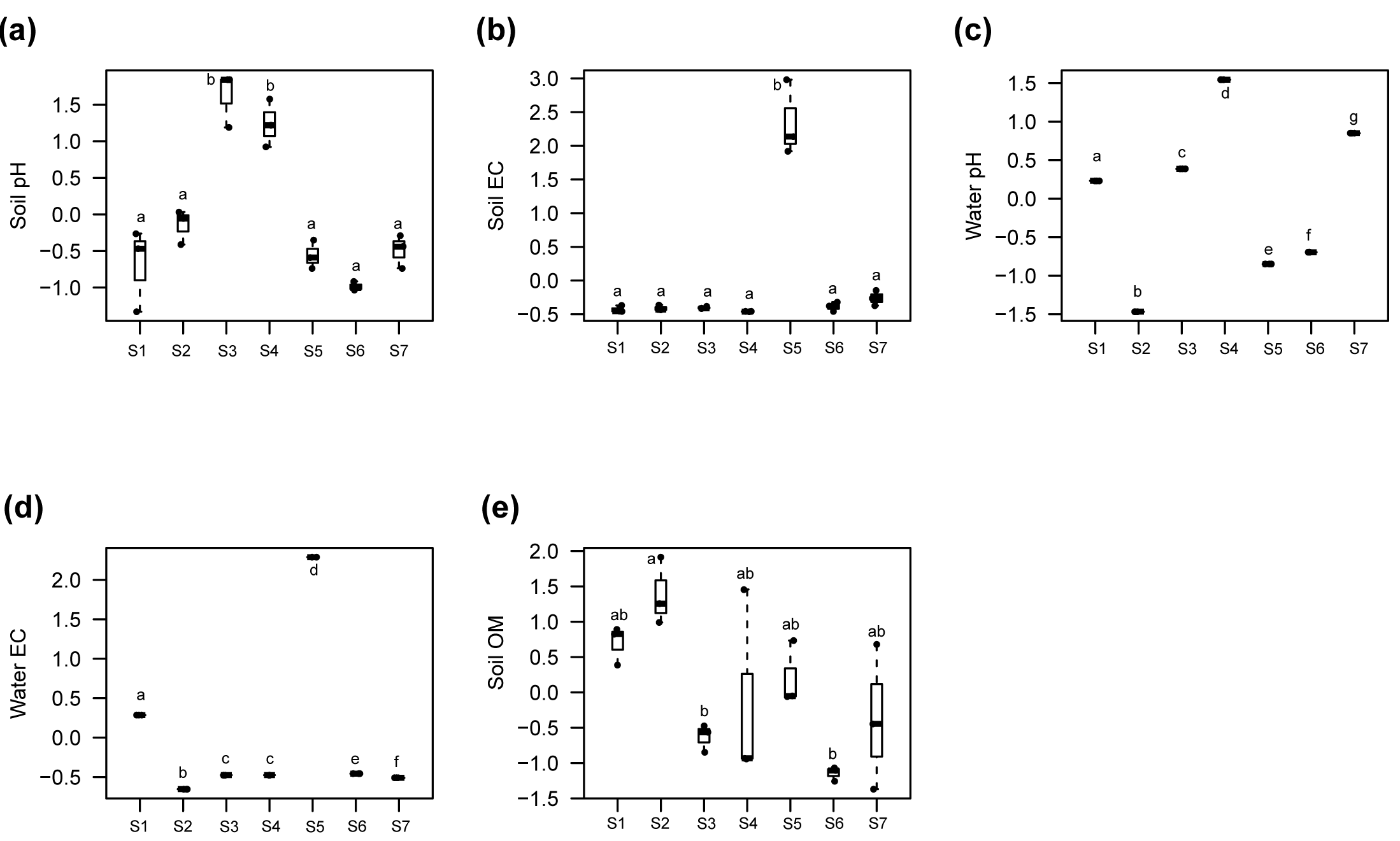
**

**Figure S4.** Box plots showing the environmental metadata under saline groundwater irrigation (a) soil pH; (b) soil electrical conductivity (EC in dS/m); (c) soil organic matter (OM); (d) irrigation water pH; and (e) irrigation water EC (EC in dS/m), measured in this study under different irrigation water types (non-saline freshwater vs saline groundwater) conditions. Statistical inference is highlighted within each panel of the plot and assessed using ANOVA analyses followed by Tukey’s HSD pair-wise test. The different alphabets denote significance at 0.05. The box spans the interquartile range (IQR; first quartile to the third) with the median indicated by a dark horizontal line, the whiskers show the 1.5xIQR. Data for each sample is also displayed with strip chart.

# **Table S1. The geographical locations of sample collection sites (NS- Non-saline freshwater and S- Saline groundwater irrigation).**

| **Site name** | **Location** | **Latitude** | **Longitude** |
| --- | --- | --- | --- |
| NS1 | Town_ceter | 24°12'58.20"N | 55°45'9.80"E |
| NS2 | Nahel_1 | 24°54'29.05" N | 55°62'08.33"E |
| NS3 | Nahel_2 | 24°52'01.5" N | 55°65'89.06"E |
| NS4 | Nahel_3 | 24°53'51.95" N | 55°60'58.67" E |
| NS5 | Al_rawda | 24°06'03.9"N | 55°32'08.6"E |
| NS6 | Seah_sharkiya | 24°12'12.66"N | 55°48'53.40"E |
| NS7 | Nabbagh | 24°18'06.0"N | 55°43'08.9"E |
| S1 | Sarooj | 24°12'08.0"N | 55°47'18.1"E |
| S2 | Nahel_1 | 24°52'02.13" N | 55°64'43.09" E |
| S3 | Nahel_2 | 24°52'25.56" N | 55°65'45.12" E |
| S4 | Nahel_3 | 24°52'02.13" N | 55°64'43.09" E |
| S5 | Nahshala | 24°24'38.49" N | 55°23'53.55"E |
| S6 | Seah_salem_east | 24°20'44.2"N | 55°27'39.9"E |
| S7 | Seah_salem_west | 24°20'45.5"N | 55°26'24.4"E |

# **Table S2. Taxonomic (phylum and order level) composition of the bacterial community retrieved from date palm roots under different irrigation sources (non-saline freshwater vs saline groundwater) in the overall and subset dataset.** Total % reads and % occurrences from overall dataset and subset are shown here. Phylum with >0.1% reads and order with >1.0% of total reads are displayed here.

| **Taxonomy** | **Overall dataset** | | **Non-saline** | | **Saline** | |
| --- | --- | --- | --- | --- | --- | --- |
|  | **Reads (%)** | **Occurrences (%)** | **Reads (%)** | **Occurrences (%)** | **Reads (%)** | **Occurrences (%)** |
| Proteobacteria | 37.4 | 34.6 | 37.6 | 33.0 | 37.1 | 36.5 |
| Rhizobiales | 16.2 | 11.4 | 17.0 | 11.0 | 15.1 | 11.9 |
| Steroidobacterales | 2.8 | 1.3 | 2.1 | 1.1 | 3.7 | 1.4 |
| Burkholderiales | 1.8 | 2.2 | 2.3 | 2.5 | 1.1 | 1.7 |
| Pseudomonadales | 1.7 | 1.3 | 1.6 | 1.2 | 1.9 | 1.3 |
| Sphingomonadales | 1.7 | 2.0 | 1.5 | 2.0 | 1.9 | 1.9 |
| Caulobacterales | 1.6 | 2.4 | 1.5 | 2.1 | 1.8 | 2.7 |
| Gammaproteobacteria Incertae Sedis | 1.5 | 1.0 | 1.7 | 1.0 | 1.3 | 0.9 |
| Enterobacterales | 1.3 | 0.3 | 2.2 | 0.3 | 0.2 | 0.3 |
| Xanthomonadales | 1.1 | 1.1 | 1.0 | 1.0 | 1.1 | 1.3 |
| Actinobacteria | 28.2 | 14.5 | 28.5 | 15.2 | 27.9 | 13.5 |
| Micromonosporales | 5.2 | 1.1 | 3.6 | 1.1 | 7.4 | 1.0 |
| Streptomycetales | 4.7 | 1.1 | 6.6 | 1.1 | 2.1 | 1.1 |
| Corynebacteriales | 4.4 | 0.7 | 3.5 | 0.6 | 5.7 | 0.8 |
| Actinomarinales | 4.4 | 1.7 | 4.9 | 1.6 | 3.8 | 1.9 |
| Microtrichales | 2.6 | 3.1 | 2.6 | 3.3 | 2.6 | 2.8 |
| Pseudonocardiales | 1.3 | 0.5 | 0.8 | 0.5 | 2.1 | 0.4 |
| Propionibacteriales | 1.2 | 1.5 | 1.6 | 1.8 | 0.8 | 1.2 |
| Firmicutes | 10.1 | 6.4 | 9.4 | 6.2 | 11.0 | 6.6 |
| Bacillales | 8.8 | 3.3 | 8.3 | 3.3 | 9.5 | 3.3 |
| Chloroflexi | 5.4 | 10.2 | 6.2 | 11.2 | 4.4 | 9.0 |
| Acidobacteriota | 4.8 | 7.9 | 4.6 | 7.9 | 5.0 | 7.9 |
| Vicinamibacterales | 1.9 | 2.7 | 2.1 | 3.0 | 1.6 | 2.4 |
| Thermoanaerobaculales | 1.2 | 1.6 | 0.9 | 1.4 | 1.6 | 1.7 |
| Bacteroidota | 4.0 | 6.6 | 3.9 | 6.7 | 4.1 | 6.5 |
| Cytophagales | 1.9 | 2.5 | 1.8 | 2.5 | 2.0 | 2.5 |
| Chitinophagales | 1.2 | 1.7 | 1.2 | 1.7 | 1.2 | 1.6 |
| Myxococcota | 2.9 | 4.3 | 3.1 | 4.6 | 2.5 | 4.0 |
| Polyangiales | 2.1 | 2.8 | 2.4 | 3.0 | 1.8 | 2.5 |
| Patescibacteria | 2.4 | 3.3 | 1.9 | 2.9 | 3.2 | 3.7 |
| Saccharimonadales | 2.2 | 2.3 | 1.6 | 1.9 | 2.8 | 2.8 |
| Planctomycetota | 1.5 | 4.6 | 1.4 | 4.5 | 1.7 | 4.8 |
| Gemmatimonadota | 0.9 | 1.8 | 0.9 | 1.6 | 1.0 | 2.1 |
| Verrucomicrobiota | 0.9 | 2.1 | 1.0 | 2.2 | 0.9 | 1.9 |
| Methylomirabilota | 0.3 | 0.4 | 0.3 | 0.4 | 0.2 | 0.4 |
| Bdellovibrionota | 0.2 | 0.6 | 0.3 | 0.9 | 0.1 | 0.4 |
| Fibrobacterota | 0.2 | 0.3 | 0.3 | 0.3 | 0.1 | 0.3 |
| Entotheonellaeota | 0.2 | 0.5 | 0.2 | 0.4 | 0.2 | 0.5 |
| Nitrospirota | 0.2 | 0.5 | 0.1 | 0.4 | 0.2 | 0.5 |
| Dependentiae | 0.1 | 0.3 | 0.1 | 0.3 | 0.1 | 0.3 |
| Latescibacteria | 0.1 | 0.3 | 0.1 | 0.3 | 0.1 | 0.3 |
| Cyanobacteria | 0.1 | 0.2 | 0.1 | 0.2 | 0.1 | 0.1 |
| Other phyla | 0.2 | 0.7 | 0.1 | 0.7 | 0.2 | 0.7 |
| Other orders | 27.1 | 50.7 | 27.1 | 51.0 | 27.2 | 50.4 |

**Table S3. Classification of OTUs based on the abundance percentages**

| **Abundance classification (% occurrences)** | **Shared OTUs** | **Non-saline freshwater irrigation unique OTUs** | **Saline groundwater irrigation**  **unique OTUs** |
| --- | --- | --- | --- |
| Abundant taxa (>1) | 13 | 0 | 0 |
| Moderate taxa (0.1 - 1) | 174 | 4 | 0 |
| Rare taxa (<0.1) | 771 | 1152 | 738 |

**Table S4.** Taxonomic lineage of top 10 root-associated bacterial shared OTUs, unique OTUs from non-saline freshwater and saline groundwater irrigation.

| **OTU_ID** | **Phylum** | **Class** | **Order** | **Family** | **Genus** |
| --- | --- | --- | --- | --- | --- |
| **Shared OTUs** | | | | | |
| OTU_3 | Actinobacteriota | Actinobacteria | Corynebacteriales | Mycobacteriaceae | Mycobacterium |
| OTU_6 | Actinobacteriota | Acidobacteriae | Actinomarinales | Uncultured | Uncultured |
| OTU_10 | Actinobacteriota | Actinobacteria | Streptomycetales | Streptomycetaceae | Streptomyces |
| OTU_13 | Proteobacteria | Alphaproteobacteria | Rhizobiales | Rhizobiaceae | Rhizobium |
| OTU_28 | Actinobacteriota | Actinobacteria | Micromonosporales | Micromonosporaceae | Micromonospora |
| OTU_143 | Actinobacteriota | Actinobacteria | Streptomycetales | Streptomycetaceae | Streptomyces |
| OTU_4 | Firmicutes | Bacilli | Bacillales | Bacillaceae | NA |
| OTU_21 | Proteobacteria | Alphaproteobacteria | Rhizobiales | Hyphomicrobiaceae | Pedomicrobium |
| OTU_9 | Firmicutes | Bacilli | Bacillales | Bacillaceae | Bacillus |
| OTU_15 | Firmicutes | Bacilli | Bacillales | Bacillaceae | Bacillus |
| **Unique OTUs in non-saline freshwater irrigation** | | | | | |
| OTU_66 | Proteobacteria | Gammaproteobacteria | Enterobacterales | NA | NA |
| OTU_30 | Actinobacteriota | Actinobacteria | Streptomycetales | Streptomycetaceae | NA |
| OTU_41 | Actinobacteriota | Actinobacteria | Streptosporangiales | Thermomonosporaceae | Actinocorallia |
| OTU_99 | Proteobacteria | Gammaproteobacteria | Enterobacterales | Erwiniaceae | NA |
| OTU_414 | Actinobacteriota | Actinobacteria | NA | NA | NA |
| OTU_98 | Proteobacteria | Gammaproteobacteria | Burkholderiales | Comamonadaceae | NA |
| OTU_163 | Bacteroidota | Bacteroidia | Bacteroidales | Bacteroidaceae | Bacteroides |
| OTU_179 | Fibrobacterota | Fibrobacteria | Fibrobacterales | Fibrobacteraceae | genus 04 |
| OTU_211 | Patescibacteria | Saccharimonadia | Saccharimonadales | NA | NA |
| OTU_236 | Proteobacteria | Alphaproteobacteria | Caulobacterales | Hyphomonadaceae | SWB02 |
| **Unique OTUs in saline groundwater irrigation** | | | | | |
| OTU_115 | Chloroflexi | Chloroflexia | Thermomicrobiales | AKYG1722 | NA |
| OTU_167 | Actinobacteriota | Acidobacteriae | Actinomarinales | NA | NA |
| OTU_186 | Acidobacteriota | Thermoanaerobaculia | Thermoanaerobaculales | Thermoanaerobaculaceae | Subgroup 10 |
| OTU_191 | Bacteroidota | Bacteroidia | Flavobacteriales | Weeksellaceae | Chryseobacterium |
| OTU_316 | Patescibacteria | Saccharimonadia | Saccharimonadales | NA | NA |
| OTU_223 | Patescibacteria | Saccharimonadia | Saccharimonadales | NA | NA |
| OTU_237 | Proteobacteria | Gammaproteobacteria | Pseudomonadales | Moraxellaceae | Moraxella |
| OTU_280 | Bacteroidota | Bacteroidia | Chitinophagales | Chitinophagaceae | Niastella |
| OTU_364 | Proteobacteria | Gammaproteobacteria | Enterobacterales | Alteromonadaceae | Rheinheimera |
| OTU_261 | Bacteria | Acidobacteriota | Blastocatellia | Blastocatellales | Blastocatellaceae |
